# Supplementary material for: Reconciling Mining with the Conservation of Cave Biodiversity: A Quantitative Baseline to Help Establish Conservation Priorities
Source: PLoS One. 2016 Dec 20;11(12):e0168348. doi: 10.1371/journal.pone.0168348 (PMC5173368; doi:10.1371/journal.pone.0168348)
Supplement: S1 Dataset — (ZIP) [file pone.0168348.s002.zip › Taxa/Serra Norte/SN_2013/N4E/Lista N4E-40.pdf]

CAVIDADE N4E-0040

Classe	Ordem	Fam/Outros	Gên/Outros	Espécie	S-U	U-S	Zon.
Arachnida	Acari	Laelapidae		sp.41		X	E
Arachnida	Amblypygi	Phrynidae	*Heterophrynus*	*longicornis*	2	X	E
Arachnida	Araneae	Araneidae	*Alpaida*	*antonio*	X	X	E
Arachnida	Araneae	Filistatidae		jovem	X		E
Arachnida	Araneae	Pholcidae	*Leptopholcus*	sp.1	X		E
Arachnida	Araneae	Pholcidae	*Mesabolivar*	sp.1	X	X	E
Arachnida	Araneae	Theridiidae		jovem	X		E
Arachnida	Araneae	Theridiosomatidae	*Plato*	sp.		X	E
Arachnida	Pseudoscorpiones	Chernetidae	*Spelaeochernes*	sp.1	X		E
Entognatha	Collembola	Entomobryidae		sp.1	X		E
Insecta	Blattodea	Blattidae		sp.5	1		E
Insecta	Blattodea	Polyphagidae		jovem	1		E
Insecta	Blattodea			jovem		X	E
Insecta	Coleoptera	Chelonariidae		sp.1		X	E
Insecta	Coleoptera	Elateridae		sp.		X	E
Insecta	Diptera	Chironomidae		sp.	X		E
Insecta	Diptera	Curtonotidae		sp.		X	E
Insecta	Diptera	Dolichopodidae		sp.	X		E
Insecta	Diptera	Muscidae		sp.	X		E
Insecta	Diptera	Phoridae		sp.		X	E
Insecta	Diptera	Psychodidae	*Sciopemyia*	*sordellii*	X	X	E
Insecta	Diptera	Tipulidae	Tipulinae	sp.	X		E
Insecta	Heteroptera	Reduviidae	Emesinae	sp.6	4		E
Insecta	Heteroptera	Reduviidae	*Phasmatocoris*	*praecellens*		X	E
Insecta	Heteroptera	Reduviidae	Reduviinae	sp.	2		E
Insecta	Homoptera	Cixiidae		sp.3	X		E
Insecta	Hymenoptera	Formicidae	*Apterostigma*	sp.		X	E
Insecta	Hymenoptera	Formicidae	*Crematogaster*	sp.		X	E
Insecta	Hymenoptera	Formicidae	*Crematogaster*	sp.1	X		E
Insecta	Hymenoptera	Formicidae	*Crematogaster*	sp.2	X		E
Insecta	Hymenoptera	Formicidae	*Gnamptogenys*	*striatula*		X	E
Insecta	Hymenoptera	Formicidae	*Nesomyrmex*	sp.1		X	E
Insecta	Hymenoptera	Formicidae	*Pachycondyla*	sp.2	X		E
Insecta	Isoptera	Termitidae		sp.		X	E
Insecta	Lepidoptera	Tineoidea		jovem		X	E
Insecta	Neuroptera	Ascalaphidae		jovem	X		E
Insecta	Neuroptera	Hemerobiidae		jovem	X		E
Insecta	Neuroptera	Myrmeleontidae		jovem	2	X	E
Insecta	Orthoptera	Phalangopsidae	*Paraclodes*	sp.	5	X	
Insecta	Orthoptera	Phalangopsidae	*Phalangopsis*	sp.	4	X	E
Insecta	Psocoptera			jovem	X	X	E
Malacostraca	Isopoda	Philosciidae		sp.		X	E
Mammalia	Chiroptera	Emballonuridae	*Peropteryx*	*kappleri*	2	2	E
